# Targeted DNA Methylation Using an Artificially Bisected M.HhaI Fused to Zinc Fingers

**DOI:** 10.1371/journal.pone.0044852

**Published:** 2012-09-11

**Authors:** Brian Chaikind, Krishna Praneeth Kilambi, Jeffrey J. Gray, Marc Ostermeier

**Affiliations:** 1 Chemistry-Biology Interface Graduate Program, Johns Hopkins University, Baltimore, Maryland, United States of America; 2 Department of Chemical and Biomolecular Engineering, Johns Hopkins University, Baltimore, Maryland, United States of America; Center for Genomic Regulation, Spain

## Abstract

Little is known about the effects of single DNA methylation events on gene transcription. The ability to direct the methylation toward a single unique site within a genome would have broad use as a tool to study the effects of specific epigenetic changes on transcription. A targeted enzyme might also be useful in a therapy for diseases with an epigenetic component or as a means to site-specifically label DNA. Previous studies have sought to target methyltransferase activity by fusing DNA binding proteins to methyltransferases. However, the methyltransferase domain remains active even when the DNA binding protein is unbound, resulting in significant off-target methylation. A better strategy would make methyltransferase activity contingent upon the DNA binding protein’s association with its DNA binding site. We have designed targeted methyltransferases by fusing zinc fingers to the fragments of artificially-bisected, assembly-compromised methyltransferases. The zinc fingers’ binding sites flank the desired target site for methylation. Zinc finger binding localizes the two fragments near each other encouraging their assembly only over the desired site. Through a combination of molecular modeling and experimental optimization in E. coli, we created an engineered methyltransferase derived from M.HhaI with 50–60% methylation at a target site and nearly undetectable levels of methylation at a non-target M.HhaI site (1.4±2.4%). Using a restriction digestion assay, we demonstrate that localization of both fragments synergistically increases methylation at the target site, illustrating the promise of our approach.

## Introduction

DNA methylation is an epigenetic modification that causes transcriptional repression. Methylation is implicated in numerous cellular processes such as X chromosome inactivation, genomic imprinting and cellular differentiation [1]–[4]. Abnormal methylation patterns have also been associated with cancer and diseases caused by deregulation of imprinted genes [5], [6]. Little is known, however, about the transcriptional effects of single CpG modifications, since there are no experimental tools to carryout single-site methylation in a genome. However, a growing body of work indicates that downregulation of expression greatly depends on the location of the methylated CpG sites within the promoter [7]. Although the promoters of silenced genes are often methylated at many sites, several transient transfection and comparative bisulfite sequencing analysis studies have shown that methylation at even a single CpG site in a promoter is sufficient to downregulate expression [8]–[11]. A targeted methyltransferase that could specifically methylate unique sites in a genome could be used to probe the effects of individual CpG modifications on transcription and on the spread of methylation. In addition, a targeted methyltransferase might be useful as a therapeutic agent for the treatment of diseases characterized by abnormal hypomethylation. Furthermore, several studies have shown that methyltransferases will accept S-adenosyl methionine analogues [12]. Coupling these analogues with a site-specific methyltransferase would allow for site-specific modification of DNA.

Many groups have biased methylation to specific DNA sequences by fusing methyltransferase enzymes to sequence-specific DNA binding proteins [13]–[19]. However, these fusion proteins still methylate away from the desired DNA sequence. This off-target activity occurs because the methyltransferase remains functional in the absence of the DNA-binding protein’s association with its cognate DNA sequence. To reduce off-target activity, methyltransferase fusion proteins have been engineered with reduced overall activity, so that a bias in methylation can be observed. However, reducing enzyme activity does not address the fundamental limitation of this strategy, and these fusion constructs still methylate at off-target sites. Furthermore, many of these studies assess the level of specificity and activity in eukaryotic cells, which contain endogenous CpG methyltransferases. This therefore limits the ability to conclusively determine the true specificity and activity of these enzymes *in vivo*. A better strategy would make methyltransferase activity contingent upon association of the DNA binding domain with its target DNA sequence. Characterization of these enzymes in *E. coli*, which lack CpG methyltransferases, rather than eukaryotic cells, will allow for the unambiguous characterization of enzymatic activity and specificity *in vivo*.

Our strategy for designing targeted methyltransferases couples the methyltransferase activity to the DNA binding protein’s association with DNA. A monomeric methyltransferase is split into two fragments that are compromised in their ability to assemble into an active heterodimeric enzyme, and each fragment is fused to a different zinc finger. The zinc fingers’ DNA binding sites flank a desired CpG site. Thus, zinc finger binding to cognate DNA sites increases the local concentration of the two attached methyltransferase fragments, encouraging the fragments to reassemble only over a desired CpG site. The association of the two fragments into an active enzyme in the absence of the flanking zinc finger binding sites is limited because the two fragments are engineered to have reduced affinity for one another or require each other for proper folding. In this manner, the strategy is akin to a protein complementation assay [20] with a specific DNA sequence mediating assembly of the active methyltransferase.

We previously demonstrated this strategy using the naturally split methyltransferase M.EcoHK31I, which methylates the internal cytosine of the 5′-YGGCCR-3′ site. We demonstrated how reduction of the fragment’s affinity for each other through truncation of one of the fragments increased the ratio of methylation at the target vs. non-target sites. The optimized construct exhibited (>50%) methylation at the target site and undetectable methylation at the non-target site under the correct expression conditions [21]. However, non-target methylation could be observed under different expression conditions. Furthermore, the M.EcoHK31I targeted cytosine residue is not a CpG site, and therefore would not be applicable for CpG methylation studies in mammalian cells.

Here we demonstrate our strategy using an artificially split M.HhaI, a CpG methyltransferase derived from M.HhaI fragments previously identified in our lab [22]. Using modeling and experimentation, we show how proper geometric configuration of the M.HhaI fragments and the zinc fingers is important for the bias and activity observed at the target site. With the proper fusion configuration of M.HhaI fragments and zinc finger proteins, we show how bias towards the target site can be increased through mutations rationally designed to reduce the association of the two fragments, through optimization of the linkers connecting the M.HhaI fragments to the zinc fingers, and through optimization of the distance between the zinc finger binding sites and the targetted methylation site. Optimization resulted in an engineered methyltransferase that methylated 50–60% of a desired the target site in *E. coli* cells with minimal levels of methylation at a non-target M.HhaI site.

## Materials and Methods

### Modeling

The structural model for M.HhaI methyltransferase was obtained from the crystal structure of the M.HhaI/DNA complex (PDB 2HR1) [23]. For target DNA sequences, straight B-DNA structures containing all the three binding sites (one M.HhaI target site and two zinc-finger binding sites) were built using the model.it web server [24].

For zinc fingers HS1 and HS2, homology models were constructed using the Rosetta comparative modeling algorithm employing zinc finger Zif268 (1AAY) as the template [25], [26]. Comparative modeling involves 1) copying coordinates from regions aligned with the template sequence, 2) a centroid pseudo-atom side-chain low-resolution building of the unaligned regions using a fragment based loop modeling protocol [27], and 3) a final all-atom high-resolution phase refinement with small backbone perturbations followed by gradient-based minimization and side-chain packing. One thousand models were generated for each of the zinc fingers and the top ranked structures based on the Rosetta standard energy function were selected. Kinks were observed in the C-terminal α-helices when these zinc finger models were superimposed on the template structure, as zinc atoms were not included during modeling. These kinks were fixed by threading the backbone of α-helices over the corresponding C-terminal α-helix from the template structure.

The final complex including zinc fingers and M.HhaI bound to the respective target sites was then assembled. The orientation of the zinc-fingers and M.HhaI at their respective binding sites was determined by aligning target DNA sequences from M.HhaI/DNA complex (2HR1) and Zif268/DNA complex (1AAY) with the straight B-DNA model.

Finally, the linker regions connecting the N-terminal and C-terminal fragments of M.HhaI to the zinc fingers were built using Rosetta kinematic closure (KIC) loop modeling algorithm [28]. The algorithm couples KIC calculations with 1) a low-resolution stage involving loop backbone minimization with side chains represented as centroids, and 2) an all-atom high-resolution stage with Monte Carlo-plus-minimization of side-chain and loop backbone dihedral angles. Methods S1 includes modeling details and command-line syntax for performing each of the aforementioned calculations using Rosetta.

### General Methods, Reagents, and Bacterial Strains

Restriction enzymes, T4 ligase, and M.HhaI were purchased from New England Biolabs (Ipswich, MA, USA) and were used according to manufacturers instructions. Oligos were purchased from Invitrogen (Carlsbad, CA, USA) and Integrated DNA Technologies (Coralville, IA, USA). Platinum® Pfx DNA Polymerase was purchased from Invitrogen (Carlsbad, CA, USA). dNTPs were purchased from Thermo scientific (Rockford, IL, USA). Agarose gel electrophoresis and PCR were preformed essentially as described previously [29].

*Escherichia coli* K-12 strain ER2267 [*F proA^+^B^+^ lacI^q^ Δ(lacZ)M15 zzf::mini-Tn10 (Kan^R^)/Δ(argF-lacZ)U169 glnV44 e14^–^(McrA^–^) rfbD1? recA1 relA1? endA1 spoT1? thi-1 Δ(mcrC-mrr)114::IS10*] was acquired from New England Biolabs (Ipswich, MA, USA) and was used for cloning and methylation protection assays.

### Plasmid and Gene Construction and Design

Plasmid pDIMN8 was derived from pDIMN7 MeND/MeCD [30]. An FspI restriction site was silently mutated within Amp^R^. Zinc finger genes were fused to M.HhaI methyltransferase gene fragments via desired length linkers using overlap extension PCR. Test sites for methylation (site 1 and site 2) were designed with an internal M.HhaI recognition site (5′-GCGC-3′) nested within an FspI restriction site (5′-TGCGCA-3′). These sites were flanked on either side by HS1 and HS2 zinc finger binding sites [31] or control DNA sequences as desired. Zinc finger recognition sites were separated from the FspI restriction site by 0, 1, 2 or 3 bp.

To facilitate changing the DNA sequences at these sites, site 1 was flanked by XmaI and EcoRI restriction sites and site 2 was flanked by AflIII and BglII sites. The BglII site was created by inserting three bp 66 base pairs downstream from the ColE1 origin of replication. The DNA at sites 1 and 2 were altered by annealing complimentary oligonucleotides encoding the desired DNA sequences. The oligonucleotides were designed such that the annealed product possessed overhangs that complemented the restriction site overhangs produced by digestion at the flanking restriction enzyme sites. Phosphorylation of the annealed oligonucleotides followed by ligation into digested vectors was used to change the sequence at sites 1 and 2.

### Methylation Protection Assays and Quantification

*In vivo* protection assays were preformed in *E. coli* strain ER2267. Frozen stocks were prepared by inoculating 10 mL of lysogeny broth, supplemented with 100 µg/µl ampicillin and 0.2% w/v glucose, with cells from a single colony. After 12–16 hrs of incubation at 37°C, 800 µl of cell culture was mixed with 200 µl of 50% v/v glycerol to create glycerol stocks, which were stored at −80°C.

To perform methylation assays, 5 µl of thawed glycerol stocks were used to inoculate 10 ml of lysogeny broth supplemented with 100 µg/µl ampicillin salt. To repress the *lac* promoter, 0.2% w/v glucose was added. To induce the *lac* and *pBAD* promoters, cultures were supplemented with 1.0 mM of IPTG and 0.0167% w/v arabinose, respectively. Experiments carried out to optimize methylation indicated that inoculation into media containing glucose, IPTG, and arabinose resulted in the highest levels of observed methylation activity. Thus, cultures contained 0.2% glucose, 1.0 mM of IPTG and 0.0167% w/v arabinose unless otherwise indicated. After 12–14 hours of incubation at 250 rpm and 37°C, plasmid DNA was isolated from the cells using QIAprep spin miniprep kit (Qiagen, Valencia, CA).

To ascertain the methylation status at sites 1 and 2 of the plasmid, plasmid DNA (500 ng) was incubated with 2.5 units of FspI and 10 units of NcoI-HF in buffer NEB4 at 37°C for 2 hours. After digestion, the DNA was electrophoresed in a 1.2% w/v agarose gel in TAE buffer at 90 V for 80 minutes at room temperature. Images were captured using the Molecular Imager XRS Gel Doc system with Quantity One software.

To quantify the percentage of plasmids methylated at each site, plasmid DNA (500 ng) was digested with 10 units of NcoI-HF and 2.5 units of FspI in buffer NEB4 at 37°C for 2 hours and half of each digested sample (250 ng) was electrophoresed in a 1.2% w/v gel for 2 hours at 90 V. Images were captured using the Gel Logic 112 Imaging System. The intensities of each of the four largest bands were determined using Carestream Molecular Imaging Software and corrected to be on a mol basis using the expected length of each DNA fragment. Percentages of methylation are based on the intensity of a given band relative to the total intensity in the lane. Each construct was tested using ≥3 independent cultures. The mean percentage is reported and the error bar represents the standard deviation (n≥3).

## Results and Discussion

Our group previously identified two M.HhaI fragments that could assemble into a functional methyltransferase enzyme in an unassisted fashion [22]. The N-terminal fragment is comprised of amino acids M.HhaI [1–240], and the C-terminal fragment is composed of amino acids M.HhaI [210–326]. Each fragment shared a common internal 30 amino acids, M.HhaI [210–240], referred to as the overlapping region. This overlapping region is analogous to the region where some natural methyltransferases are split or circularly permuted [32].

Nomura and Barbas reported that fusion of one zinc finger to the N-terminus of M.HhaI[1–240] and a second zinc finger to the C-terminus of M.HhaI[210–240] resulted in a targeted methyltransferase [33]. However, our analysis of their engineered enzyme using more definitive assays showed that it methylates target and non-target sites with the same low efficiency [30]. Nevertheless, we imagined that our fragments might be converted to a targeted methyltransferase if we (1) fused the fragments to zinc fingers in the correct orientation relative to the target DNA sequence, (2) reduced the fragments ability to assemble in an unassisted fashion through mutations designed to reduce the fragments’ affinity for each other, (3) optimized the linkers connecting the fragments to the zinc fingers, and (4) optimized the number of bases separating the zinc finger binding sites from the M.HhaI recognition site.

### Initial Studies

**Figure 1:**
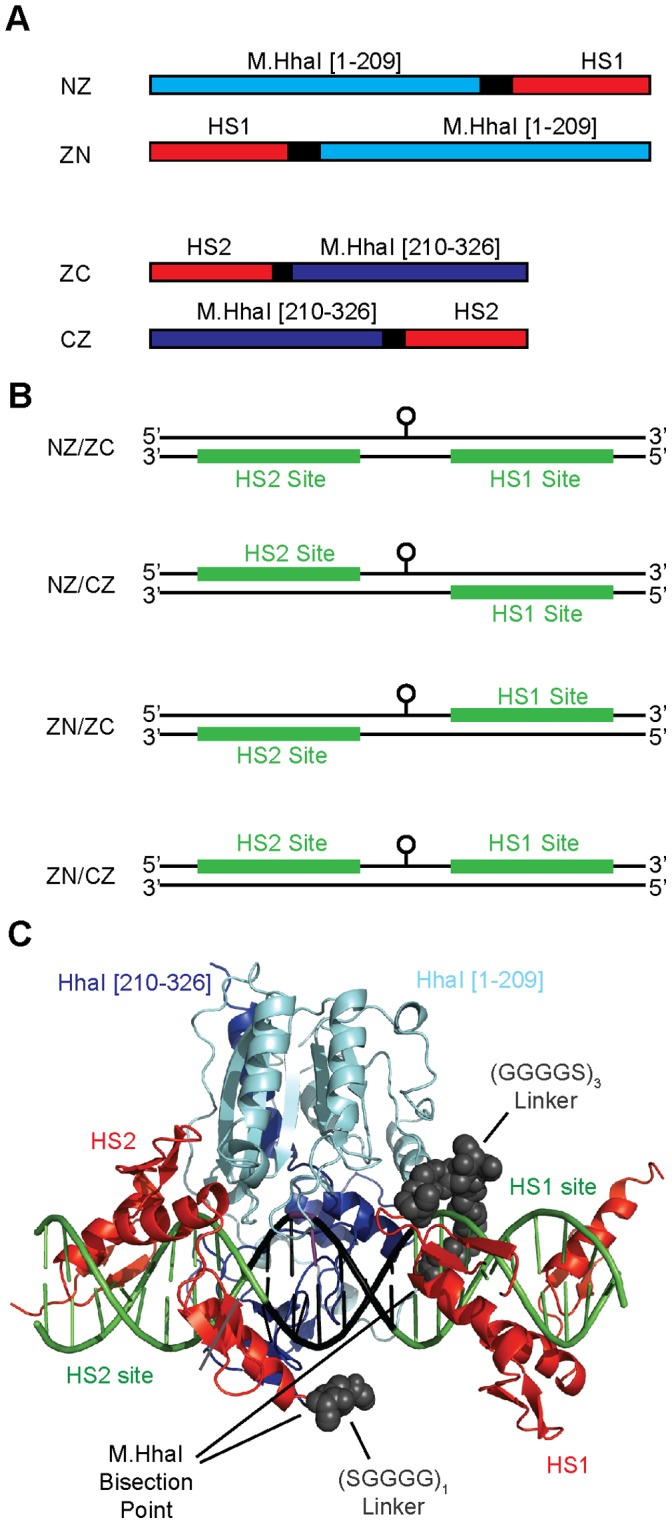
Schematic depictions of sequences and nomenclature of modeled protein/DNA complexes. (**A**) Sequences of zinc fingers fused to fragments of M.HhaI methyltransferase. Numbers in brackets correspond to the amino acid numbers. Black segments correspond to linker sequences. (**B**) The orientation of the zinc finger binding sites relative to the intended methylation target site (the circle). The orientations depicted are the ones that would position the indicated protein pairs over the targeted CpG site. (**C**) Molecular model of a particular NZ/CZ construct containing the indicated linkers. The base to be methylated is indicated in purple. Models of other complexes can be found in Figure S1.

In principle, each methyltransferase fragment could be fused to a zinc finger at the fragment’s N- or C-terminus (Figure 1A); combining these fusion variants creates four distinct zinc finger/methyltransferase fragment fusion topologies. We have previously shown that a particular ZN/CZ fusion pair (see Figure 1A for nomenclature) designed by Nomura and Barbas [33] exhibits low-level, non-specific methylation of M.HhaI DNA sites *in vivo* [30]. In contrast, our initial tests of the NZ/ZC fusion pair displayed some bias towards a target flanked by the zinc finger binding sites.

We next desired to model all four combinations of fusion pairs to predict the optimal combination for fragment reassembly at the target site and to estimate the linker lengths that would be required to connect the zinc finger and the methyltransferase fragments. However, the presence of the 30 amino acid overlapping region on both fragments complicated the modeling. We wondered if this region could be removed from one of the two fragments without compromising activity. Using the NZ/ZC construct, we conducted a set of experiments designed to probe the importance of the common 30 amino acids present in both the N-terminal fragment and C-terminal fragment. These experiments revealed that when the fragments are fused to zinc fingers, the 30 amino acids could be removed from the N-terminal fragment (but not from the C-terminal fragment) without reducing methyltransferase activity. The fragment pair M.HhaI [1–209] and M.HhaI [210–326], which lacks any overlap in sequence, formed the basis for all subsequent experiments.

### *In silico* Modeling Illustrates the Spatial Constraints of a Functional M.HhaI Heterodimeric/zinc Finger Fusion Protein with Targeted Activity

We used *in silico* modeling to predict the structures of the four possible combinations of M.HhaI fragments and zinc fingers bound to DNA. Each of the four pairs of fusion combinations required a particular placement of the zinc finger binding sites relative to the internal CpG site (Figure 1B). Other orientations of the zinc finger binding sites relative to the internal CpG site would present one or both methyltransferase fragments away from this targeted cytosine. For each of the four configurations depicted in Figure1B, we produced two models in which the methyltransferase was positioned to methylate either the top or the bottom strand relative to the bound zinc fingers (Figure S1). Modeling assumed straight B-DNA structure and thus does not capture any distortions of the DNA that may or may not be induced by the binding of the fusion proteins to DNA.

Modeling predicted that fusion of the zinc fingers to the fragments at the bisection site (i.e. configuration NZ/ZC) would best position the fragments in an orientation capable of reassembling and therefore methylating a targeted CpG site (Figure 1C). We judged this pair as optimal because it required the shortest linkers connecting the zinc fingers to the methyltransferase fragments. All configurations other than NZ/ZC required linkers that would need to circumvent long distances around the DNA or methyltransferase domains and connect residues separated by at least 40 Å (Figure S1). Although one could conceivably use very long, flexible linkers to traverse these long distances, we reasoned that such constructs would do a poorer job of increasing the local concentration of the two fragments at the target site.

The NZ/ZC model indicated that the linker connecting the N-terminal fragment and its respective zinc finger would need to be longer than that connecting the C-terminal fragment and its zinc finger. The NZ/ZC models also suggested that methylation of one of the strands may be less favorable since it requires the linkers to cross (Figure S1). The models were consistent with our initial experimental results and provided a rationale for why the NZ/ZC fusion pair, but not the ZN/CZ pair, exhibited some bias for methylating the target site. The models also supported our hypothesis that methylation could be biased towards a target site via a DNA-targeted reassembly method that works by increasing the local concentration of the fragments at the target site.

### Plasmid and Restriction Enzyme Protection Assay Design

**Figure 2:**
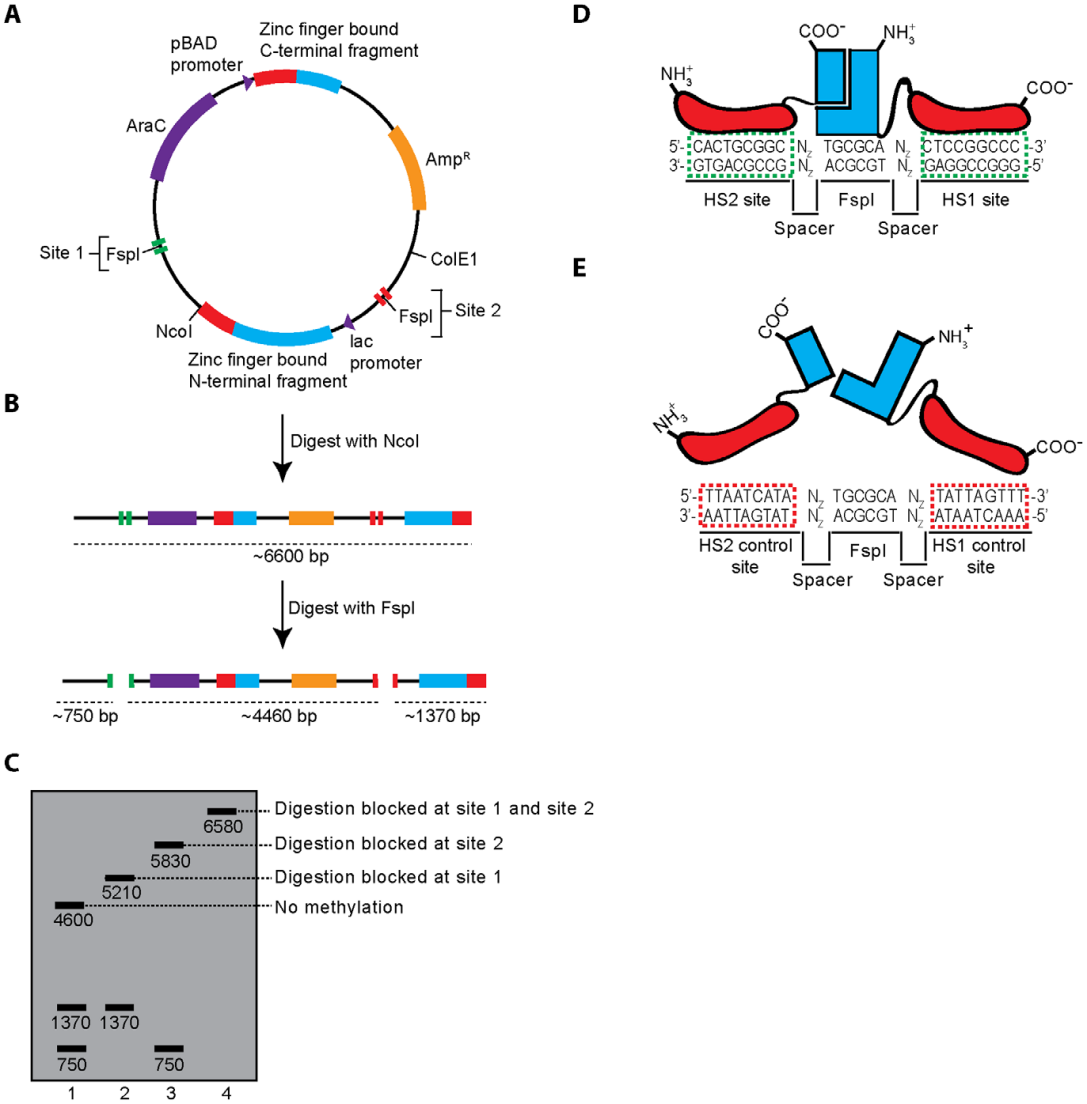
A schematic of the restriction enzyme protection assay for targeted methylation. (**A**) A single plasmid, pDIMN8, encodes genes for both methyltransferase fragment-zinc finger fusion proteins, as well as two sites for assessing the degree of targeted methyltransferase activity. Expression of both protein fragments was induced in ER2267 cells and plasmid DNA was isolated. (**B**) Plasmid DNA was linearized by NcoI-HF digestion and incubated with FspI, an endonuclease whose activity is blocked by methylation. In the absence of methylation, the plasmid is digested twice by FspI and once by NcoI-HF as shown. (**C**) Methylation at one or both of the FspI containing sites creates unique digestion patterns as assessed by agarose gel electrophoresis. Unique bands are diagnostic of no methylation (∼4600 bp), methylation at site 1 (∼5210 bp), methylation at site 2 (∼5830 bp), or methylation at both sites (∼6580 bp). (**D**) A schematic of the functional methyltransferase at a target site. Zinc finger/DNA recognition mediates methyltransferase assembly. (**E**) This assembly is designed not to occur at the non-target control site, which lacks zinc finger binding sites.

We placed the genes encoding the NZ/ZC fragment pairs in a plasmid under separate inducible promoters (Figure 2A). These genes also encoded different length peptide linkers connecting the zinc fingers and the methyltransferase fragments. For assessing methylation levels, the plasmid also contained two M.HhaI test sites (5′-G**C**GC-3′) that were nested within FspI sites (5′-TG**C**GCA-3′). FspI digestion is blocked by ^5m^C methylation at the first cytosine in the recognition sequence [34]. The plasmid also contained a unique NcoI site, so that linearization by NcoI, along with incubation with FspI and agarose gel electrophoresis could be used to distinguish between the four possible methylation states of these two sites (Figure 2B,C). The two test sites were flanked by HS1 and HS2 zinc finger binding sites (Figure 2D), control sequences (Figure 2E), or combinations thereof. Various length spacer nucleotides separated the FspI site and these sequences. The *in vivo* methyltransferase activity assay was preformed by culturing ER2267 cells containing these plasmids in the presence or absence of the inducers for methyltransferase fragment expression. Plasmids were isolated, incubated with NcoI and FspI, and the digestion patterns analyzed by agarose gel electrophoresis.

### Reduction of Off-target Activity through Serial Truncation of the C-terminal Fragment

We hypothesized that methylation at non-target sites resulted from the reassembly of the M.HhaI [1–209] and M.HhaI [210–326] fragments in the absence of the zinc finger binding sites, much like the M.HhaI [1–240] and M.HhaI [210–326] fragments that can assemble in an unassisted fashion [22]. We attempted to improve the bias for the target site through mutations designed to reduce the affinity of the two methyltransferase fragments for one another.

The C-terminal α-helix of M.HhaI is located on the C-terminal fragment and interacts with a set of β-strands located on the N-terminal fragment. Together, the helix and β-strands comprise part of the Rossmann fold in M.HhaI [35]. We hypothesized that truncation of the C-terminal α-helix might disrupt this interaction by either reducing the overall stability of the C-terminal fragment or by simply reducing the surface area of the protein-protein interface. Thus, truncation of the C-terminal helix was designed to prevent fragment reassembly when zinc fingers were not bound to their target sites, reducing off-target methylation. Zinc finger binding would facilitate the two fragments’ assembly at the target site.

**Figure 3:**
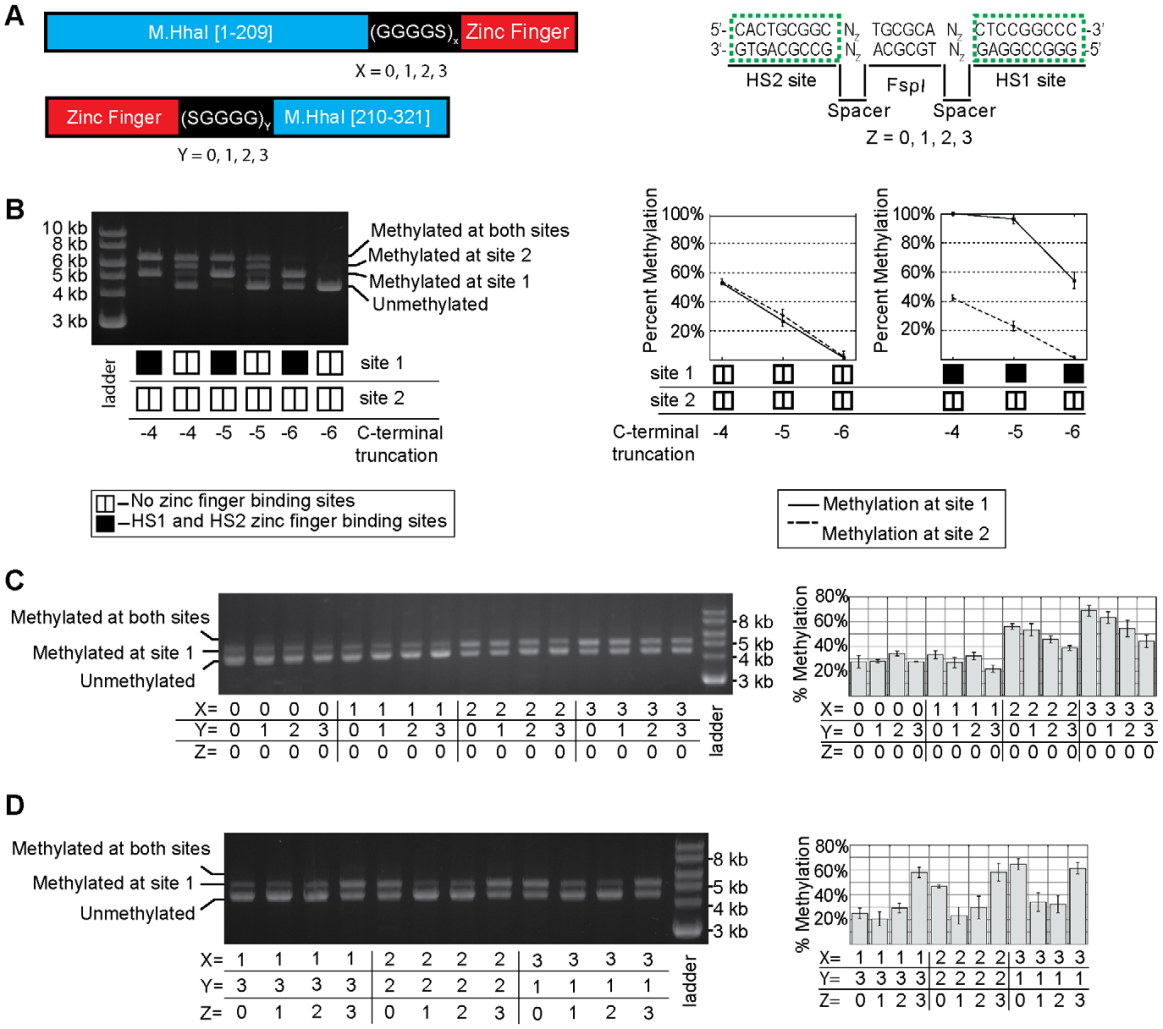
The effect of C-terminal truncation, linker lengths, and target site spacing on methyltransferase activity. (**A**) A schematic of the protein fusions and target DNA sequences indicating the variability in linker length and DNA spacing tested. The linkers connecting the zinc fingers to the N- and C-terminal fragments were varied in 5 amino acid increments (from 0 to 15 amino acids), and combined iteratively. The bases separating the FspI site from the zinc finger binding sites were also varied (0,1,2,3 bases on each side). (**B**) Truncation of the C-terminus of the C-terminal fragment (indicated in units of amino acids) decreases off-target activity at the methyltransferase. In this experiment X = 3, Y = 1 and Z = 0. The nature of the DNA at site 1 and site 2 (whether a target or non-target site) is depicted at the bottom of the figure and graph. Constructs in which the C-terminus of M.HhaI was truncated by 6 amino acids were used to determine the effect of (**C**) linker length and (**D**) target site spacing on methyltransferase activity at the target site. The percent methylation at the target site are indicated in the graphs. All graphs show the mean and the error bar represents the standard deviation of the analysis of plasmid DNA from n ≥3 independent cultures.

Based on the model of NZ/ZC, we used a long linker to connect the N-terminal fragment with HS1 and a short linker to connect HS2 with the C-terminal fragment (i.e. X = 3 and Y = 1 in Figure 3A). No spacer nucleotides were placed between the FspI site and the HS1/HS2 binding sites (i.e. Z = 0 in Figure 3A). As shown in Figure 3B, the progressive deletion of 4 to 6 amino acids from the C-terminus of the C-terminal fragment resulted in the maintenance of a relatively high level of methylation at the target site (>50%) but a severe reduction in methylation at the non-target site. Digestion of truncation variants with methylation-sensitive endonuclease HhaI, corroborated our observation that a heterodimeric enzyme with a C-terminal deletion of 4 or 5 amino acids maintained appreciable levels of off-target methylation (Figure S2A). With 6 amino acids deleted, we observed 53±3% methylation of the target site and 1.4±2.4% methylation at the non-target site (Figure 3B) Methylation was not apparent at any other M.HhaI site based on restriction digest protection assays with HhaI (Figure S2A), though the assay is not as sensitive for methylation as the assay with FspI at the non-target site due to the large number of HhaI sites. Similarly, HhaI digestion of genomic DNA failed to provide any evidence of off-target methylation with our optimal construct (Figure S2B), though significant off-target methylation would need to occur for this assay to detect methylation on the chromosome.

Bisulfite sequencing confirmed that methylation at the target site caused protection from restriction enzyme digestion (Figure S3A). No methylation could be detected at the non-target site by bisulfite sequencing (Figure S3B). As our model predicted, methylation occurred preferentially on one strand of the target site. The methylation on this strand was on the order of 50%; the other strand, which would require the linkers to cross, may have trace amounts of methylation. However, the cytosine trace peak on this strand is not much higher than the underlying background (Figure S3A).

**Figure 4:**
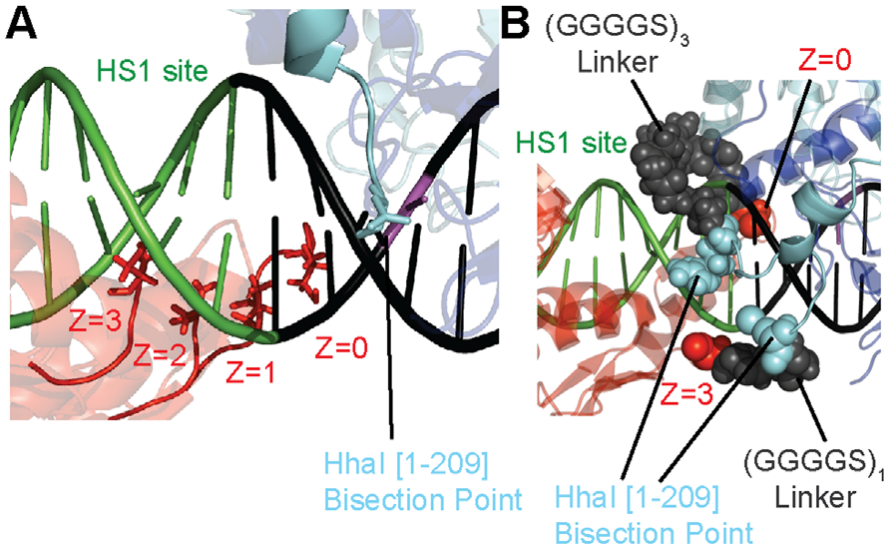
Molecular modeling explains how an increase in target spacing can reduced the required protein linker length. (**A**) HS1 zinc fingers are bound to DNA with a target site spacing of Z = 0,1,2, or 3. Note that at Z = 3, the zinc finger is actually closer to the bisection point of the N-termini than at Z = 0. (**B**) A model demonstrating that a five amino acid linker is sufficient to connect zinc finger HS1 bound at Z = 3. In contrast, a longer amino acid linker is required to circumvent the DNA backbone at Z = 0. This model provides an explanation for the pattern of target site methylation observed in Figure 3D.

### The Linker Length’s Effect on Methylation at the Target Site is Consistent with the Model

We next sought to investigate and optimize the length of the amino acid linkers connecting the M.HhaI fragments to their respective zinc fingers. Our previous work with targeted split methyltransferases indicated that linker length can affect enzymatic activity at the target site [21]. Using overlap extension PCR, we created N-terminal and C-terminal fragments that were fused to zinc fingers by linkers of 0, 5 10 and 15 amino acids (Figure 3A). In all constructs, the C-terminal M.HhaI fragment had its last 6 amino acids removed. All N-terminal fragment linker variants were then crossed with all C-terminal linker variants and tested for methylation activity at the target and non-target site (Figure 3C). The target site lacked spacer nucleotides (Z = 0).

**Figure 5:**
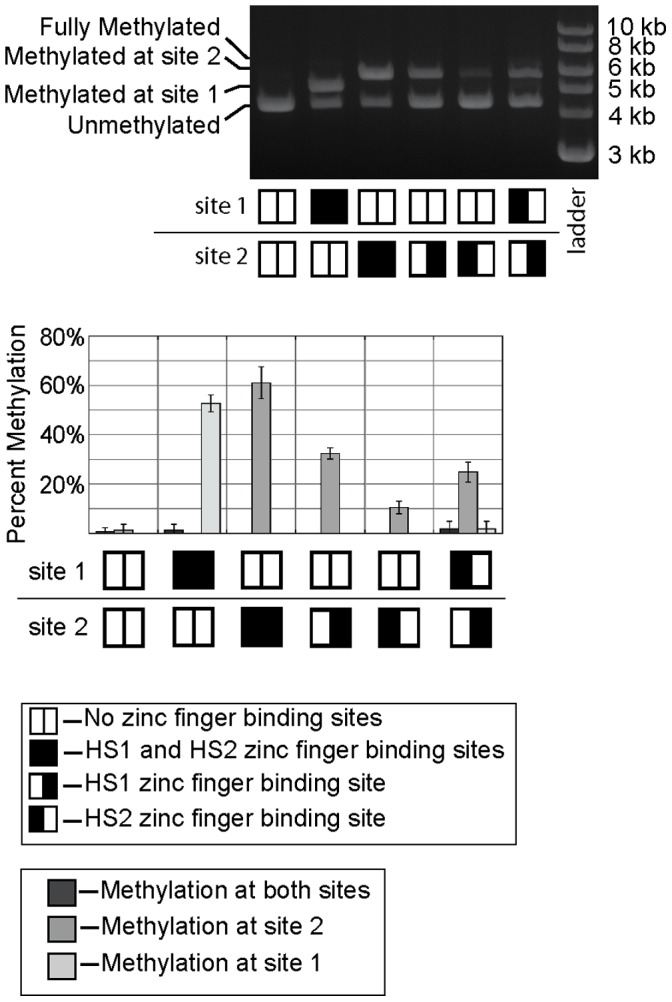
The contribution of each zinc finger binding site toward observed, targeted DNA methylation. Methylation was assessed as in Figure 2. In this experiment, the C-terminal fragment of M.HhaI is truncated by 6 amino acids, X = 3, Y = 1, and Z = 0. Methyltransferase activity was assessed with and without target sites present. Moving the target site from site 1 to site 2 did not have a large effect on activity. Target half sites (in which either the HS1 or HS2 binding sites were removed) allowed assessment of the contribution of each zinc finger on methylation activity at the target site. The sum of the methylation observed on each half site (43±5%) was less than methylation at the full target site (61±6%). The methylation observed with two distal half sites (<30%) was also less than that observed with the complete target site.

All constructs retained bias for methylation at the target site. We observed a reduction in methylation at the target site for shorter amino acid linkers connecting the N-terminal fragment with its respective zinc finger protein. Conversely, in the context of long N-terminal linker, an increase in the length of the linker connecting the C-terminal fragment and its respective zinc finger resulted in a decrease in methylation at the target site.

**Figure 6:**
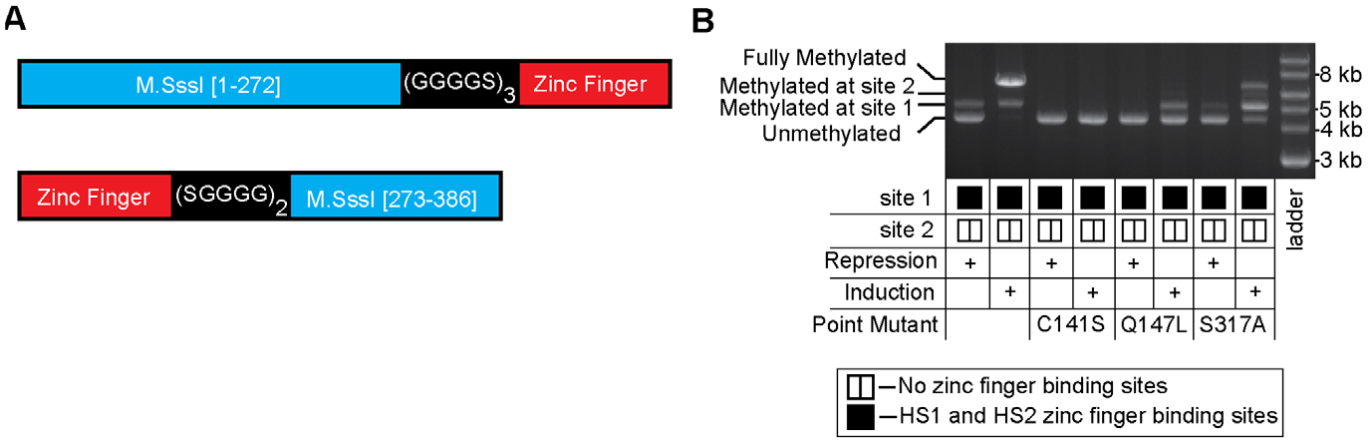
M.SssI can be converted into a targeted heterodimeric methyltransferase. (**A**) A schematic showing the sequence of the M.SssI fragments fused to zinc fingers via flexible linkers. (**B**) A restriction enzyme protection assay showing the split enzyme constructs possess a bias for methylation at the target site. Plasmids were isolated from strains grown under conditions that either repress or induce expression of the two fragments. Plasmid DNA was assayed for methylation as in Figure 2. The activity of these fusion heterodimers was attenuated by the indicated point mutations known to decrease enzyme activity in wild-type M.SssI.

The relationship between linker length and activity at the target site can be explained by our model of the NZ/ZC/DNA complex. As shown in Figure 1C, the optimal 15 amino acid linker, connecting the HS1 zinc finger to the N-terminal fragment, is found to wrap around the DNA backbone. The figure shown is one of two equally possible confirmations that can be adopted by the N-terminal linker. A longer linker is required because the N-termini of the zinc finger and the bisection point of the N-terminal methyltransferase fragment are located on opposite sides of the DNA. Shortening the linker reduces the probability of interaction between the N-terminal M.HhaI fragment with the C-terminal M.HhaI fragment upon HS1 zinc finger binding, thereby reducing methylation at the target site. On the other hand, the close proximity of the N-terminus of the C-terminal M.HhaI fragment and HS2 zinc finger indicates that a short linker would be sufficient between these two domains. However, it would be less entropically favorable for a longer flexible linker between these domains to assist in the assembly of an active enzyme. An overly long linker enables the C-terminal fragment to explore more space upon zinc finger binding, compromising the increase in local concentration of the C-terminal fragment gained by zinc finger binding.

### The Orientation of the Zinc Finger Binding Sites Relative to the Methylation Site Modulates Targeted Methylation

We next sought to characterize the effect of adding bases between the zinc finger binding sites and the FspI site (i.e. varying Z in Figure 3A). The addition of bases both increases the distance in the DNA sequence and rotates the zinc finger binding sequence around the DNA with respect to the central CpG site. Due to this rotation, the addition of bases can potentially increase or decrease the required length of the linker joining the methyltransferase fragment and the zinc finger.

To test this idea, we used three sets of linker variants in which the sum of the number of linker residues was kept constant (X = 1/Y = 3, X = 2/Y = 2, and X = 3/Y = 1). A constant sum total of linker residues helps illustrate that total linker length does not determine enzymatic activity at the target site. For each set of linker variants, we added 0, 1, 2 or 3 bp to both sides of the FspI site and tested methyltransferase activity as before. All constructs retained some methylation at the target site and minimal methylation at the non-target site; however, the length of the spacer DNA modulated activity in a complex manner (Figure 3D).

The NZ/ZC/DNA model provides a rationale for the observed behavior. For X = 1/Y = 3/Z = 0, methylation at the target site remains low because the linker between the N-terminal M.HhaI fragment and its respective zinc finger is too short. However, methylation at the target site increases to 58% at the highest spacer length (Z = 3) because the linker between these domains no longer needs to wrap around the DNA backbone (Figure 4A). When Z = 3, modeling indicates that a five amino acid linker (X = 1) between the two domains is sufficient for fragment reassembly at the target site (Figure 4B).

For linker combinations that possess high target site methylation with Z = 0 (i.e. X = 2/Y = 2 and X = 3/Y = 1), the addition of 1 or 2 bp reduces methylation at the target site. However, the addition of 3 bases restores target site methylation to their Z = 0 levels (Figure 3D). The initial reduction of activity with the addition of 1 or 2 bp can be explained by the rotation of the zinc fingers further around the DNA such that the linkers have to span even longer distances for reassembly to occur. However, modeling predicts that with the addition of 3 bp, the linker can now circumvent the DNA in the opposite direction and the distance can now be spanned by the 10 or 15 amino acid linkers connecting the N-terminal fragment and HS1 (Figure 4B).

### Zinc Finger Mediated Localization of Both M.HhaI Fragments has a Synergistic Effect on methylation Targeting

We desire targeted methyltransferases that require the binding of both zinc finger domains for methyltransferase activity. However, all linker length and spacer DNA variants tested (Figure 3C,D) retained some bias for methylating the target site, despite our models’ prediction that some variants have insufficient length linkers to allow target site reassembly. This suggests that some of the methylation observed at the target site may occur without binding of both zinc fingers. In other words, the bias for methylation at the target site may occur in part through localization of only one of the two fragments via its zinc finger domain, followed by a reassembly of M.HhaI that is independent of a second zinc finger binding event.

To test this hypothesis, target “half sites” were constructed with either the HS1 or the HS2 zinc finger binding site (Figure 5). These experiments were conducted with a construct containing a high degree of specificity and activity for the full HS1/HS2 site (i.e. X = 3, Y = 1, Z = 0). The amount of methylation at the target and non-target sites was assessed as before. Removal of either (but not both) of the zinc finger binding sites reduced, but did not eliminate methylation at the target site (Figure 5). Removal of the HS1 binding site was more detrimental to methylation activity at the target site than removing the HS2 site, indicating that localizing only the N-terminal M.HhaI fragment via zinc fingers was more effective for targeting methylation than localizing only the C-terminal fragment via zinc fingers. This result may be explained by the fact that the target recognition domain (TRD) is present on the C-terminal fragment. Thus, the C-terminal fragment likely possesses greater inherent affinity for the methylation target site than the N-terminal fragment. In other words, the N-terminal fragment has a greater need for fusion to the zinc finger in order to localize it to the target site. This result is unlikely to be explained by differences in the DNA binding affinity of the two zinc finger proteins. HS1 and HS2 have similar dissociation constants for their target sites (35 nM and 25 nM, respectively) [31].

Although this experiment revealed a shortcoming of our current optimized, split M.HhaI, it also provides evidence for the advantages of targeting methyltransferases using our split enzyme strategy. The level of target site methylation observed at a CpG site flanked by both zinc finger binding sites (61±6%) exceeds the sum of the methylation observed at the half sites (43±5%) (Figure 5). This synergy (i.e. the observed activity at the intact target site is greater than the sum of activity observed at the individual binding sites), is caused by the proximity of zinc finger binding sites and is precisely what our split enzyme system was designed to achieve. We also confirmed that placing the two zinc finger sites at distant locations on the same plasmid cannot provide the same level of targeted methylation observed by placing both zinc finger sites at one target site (Figure 5).

### M.SssI can be Converted into a Heterodimeric/zinc Finger Fusion Enzyme, whose Activity is Biased Towards a Desired Target Site

We were interested in assessing whether other monomeric methyltransferases could be bisected and fused to zinc finger proteins to create targeted methyltransferase. Specifically, we were interested in bisecting M.SssI, a prokaryotic methyltransferase that recognizes and methylates the cytosine of any 5′-CG-3′ site. A targeted methyltransferase derived from M.SssI would, in theory, make it possible to target any CpG site, rather than just 1/16 of the possible CpG sites that could be methylated by our engineered M.HhaI enzyme (which recognizes 5′-GCGC-3′).

We used a CLUSTALW alignment to identify a site within M.SssI that was similar to the split site of M.HhaI [36], [37]. We fused the zinc finger proteins HS1 and HS2 in the same NZ/ZC orientation as our targeted M.HhaI fusion proteins using 15 and 10 amino acid linkers, respectively. (Figure 6A). These constructs were tested for methylation specificity in an analogous fashion to that illustrated in Figure 2. Methylation activity was assessed under conditions shown to either induce or repress expression of the methyltransferase fragments. Upon induction, the fusion constructs were very active and, although some bias towards the target site was apparent, the high activity prevented the observation of the extent of this bias (Figure 6B). Slaska-Kiss et al very recently demonstrated that M.SssI is amenable to protein fragment complementation at select sites; however, targeted methylation was not demonstrated [38].

We used site-specific mutagenesis to reduce methyltransferase activity in order to reveal the inherent bias of the construct (Figure 6B). Mutating the active site cysteine, C141S, has been shown to reduce the activity of M.SssI enough to reveal biased methylation activity upon M.SssI fusion to triple helix forming nucleotides [39]; however, in the context of our bisected enzyme, this mutation completely eliminated activity *in vivo* (Figure 6B). On the other hand, both the Q147L and S317A mutations, which are known to reduce M.SssI’s DNA binding affinity by 12-fold and 3-fold respectively [40], reduced but did not eliminate the activity of our bisected M.SssI, revealing the extent of our enzymes methylation bias (Figure 6B). The relative activity of the two mutants was consistent with the reported relative effect of the mutations on M.SssI affinity for DNA.

### Conclusions

We have demonstrated that bisected M.HhaI and M.SssI enzymes, when fused to zinc fingers in the proper orientation, can target methylation to a desired sequence flanked by the respective zinc finger binding sites. Our modeling and experiments have elucidated some of the design principles for constructing a targeted methyltransferase using this strategy. The orientation of methyltransferase fragments relative to each other and to DNA affect the activity at the target site. Mutations designed to reduce the interaction between fragments can improve targeting of the methyltransferase. With the proper linker length, spacing between zinc finger binding and methylation sites, and expression conditions, such constructs can methylate a desired target with high efficiency (50–60%) with levels of off-target methylation at or below the limit of detection. Part of the targeting arises from the synergistic effect of localizing both fragments to the desired site, which supports our hypothesis of how bisected enzymes could better target methylation.

However, some of the bias for methylation at the target site likely arises from zinc finger mediated localization of only one of the two fragments. Thus, although binding of both zinc finger domains increases target site methylation, such methylation does not yet *require* binding of both zinc finger domains. We believe this limitation arises because the individual N- and C-terminal fragments (particularly the C-terminal fragment) retain some affinity for the 5′-GCGC-3′ site and, perhaps, retain sufficient affinity for each other. We next intend to test these hypotheses experimentally in a manner that is guided by our computational model.

### Supporting Information

Supporting information is available online: Supplementary Figures S1–3, Methods S1 and Models S1.

## Supporting Information

Figure S1 (**A–D**) **Models for four possible fusion combinations of M.HhaI fragments and zinc fingers bound to DNA.** For each of the four configurations, models were constructed with the methyltransferase positioned to bind the top or the bottom strand relative to the bound zinc fingers (columns 1 and 2). The ‘X’ and ‘Y’ labels indicate the location of the zinc finger and methyltransferase termini that need to be connected via a peptide linker in order for the zinc finger and M.HhaI domains to be bound to DNA as labeled. The ‘X’ label present on zinc finger HS1 termini should be fused to the ‘X’ label on the termini of M.HhaI [1–209]. The ‘Y’ label present on zinc finger HS2 termini should be fused to the ‘Y’ label on M.HhaI [210–326]. For linear representations of the fusion genes and binding sites see Figures 1A and 1B.
(TIF)

Figure S2 **HhaI protection assay of C-terminal truncation variants shown in** **Figure 3A** **.** (A) Analysis of plasmid DNA. HhaI endonuclease activity is blocked by methylation and one band is indicative of methylation and protection at the target site (site 1). Other, larger bands are indicative of off-target methylation. There are 36 HhaI recognition sites on pDIMN8. Therefore, this assay cannot detect all of the off-target methylation as some bands indicative of off-target methylation may be obscured by other bands in the same lane and some may be too small to observe by this method. (B) Analysis of genomic DNA using FspI digestion. Chromosomal DNA was isolated from cells containing engineered M.HhaI constructs with a 6 or 4 amino acid C-terminal truncation where X = 3, Y = 1, Z = 0. The cells were grown under conditions known to repress or induce methyltransferase fragment expression (see Materials and Methods and Figure 3). The K12 chromosome has over 2000 FspI restriction sites; thus, individual digestion products are not resolvable. For the 6 amino acid-deletion variant this digestion pattern for chromosomal DNA isolated from cells with induced or repressed expression of the engineered methyltransferase is indistinguishable, indicating little to no methylation. However, for the 4 amino acid-deletion variant, induction of the engineered methyltransferase, shifts the digestion pattern toward higher molecular weight bands, which is indicative of some chromosomal methylation. As a control, chromosomal DNA treated with M.HhaI in vitro is protected from FspI digestion. The results show that our targeted methyltransferase (with the 6 amino acid truncation) causes little to no methylation of the chromosome.
(TIF)

Figure S3 **Bisulfite analysis of both strands of the target site and the non-target site.** Bisulfite analysis of both strands of (A) the target site and (B) the non-target site. Bisulfite treatment followed by PCR amplification converts unmethylated cytosine bases to thymidine bases. Methylated cytosine residues are protected from such a conversion. The sense strand is defined as the top strand of the target and non-target sites shown in Figure 2D and 2E; the antisense strand is the bottom strand in these figures. Sequenced plasmid DNA, which was not bisulfite-treated is shown in column 1 row 1 of each panel. For both panels, the chromatogram of the antisense strand (column 2 row 1) is the computer-generated reverse complement of the chromatogram in column 1 row 1. DNA in rows 2–6, was treated with the bisulfite reagent, amplified and sequenced as described in Methods S1. The plasmid tested was X = 15, Y = 5, Z = 0 (see Figure 3). Rows 2 and 3 show sequencing results for bisulfite-treated unmethylated and methylated control DNA. Rows 4–6 show sequencing results for bisulfite-treated plasmid DNA from three independent cultures. The chromatograms for the following samples were converted to the reverse complement to simplify the comparison (target site, column 1, rows 4–6).
(TIF)

Methods S1 Supplementary methods section includes modeling details and command-line syntax, HhaI restriction assay, chromosomal restriction assay, and bisulfite sequencing.
(DOCX)

Models S1 PDB files of all models in a.zip file format.
(ZIP)
