# Correction: Targeted DNA Methylation Using an Artificially Bisected M.HhaI Fused to Zinc Fingers

**DOI:** 10.1371/annotation/b1f48851-2dd7-438f-938e-6bac0b4d8c94

**Published:** 2014-01-29

**Authors:** Brian Chaikind, Krishna Praneeth Kilambi, Jeffrey J. Gray, Marc Ostermeier

There is an error in Figures 1-4, Figure S1 and Model S1. The authors made an error in their model which resulted in a shift in where the HS1 and HS2 zinc fingers are predicted to bind. Please view the correct Figures 1-4, Figure S1 and Model S1 here:

Figure 1: 

**Figure:**
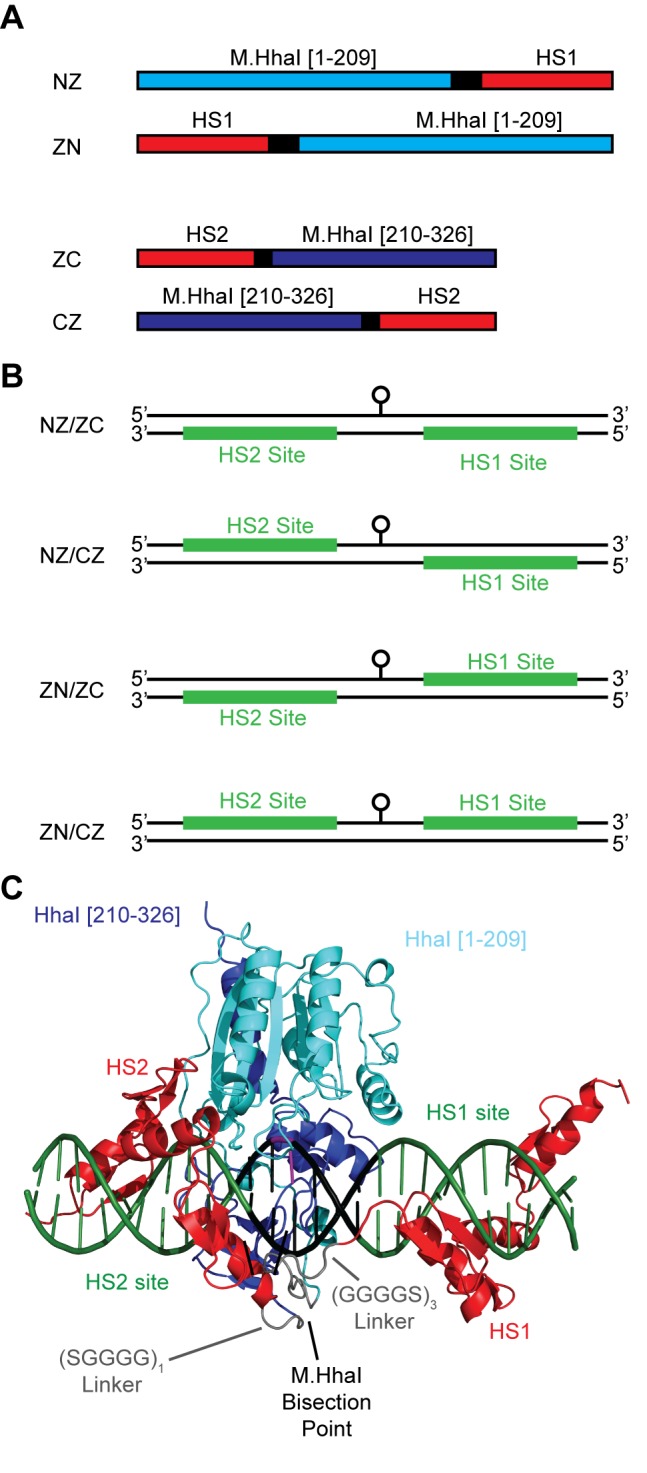


Figure 2: 

**Figure:**
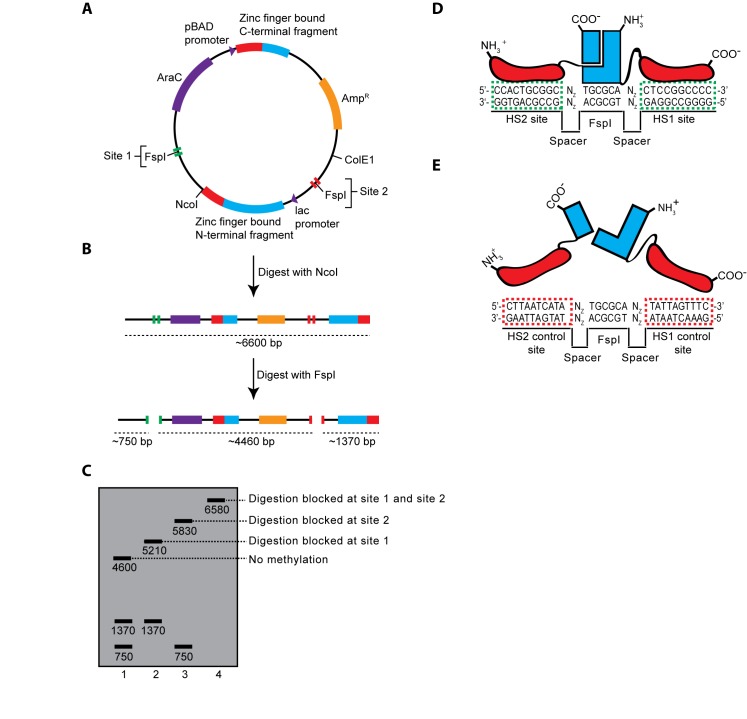


Figure 3: 

**Figure:**
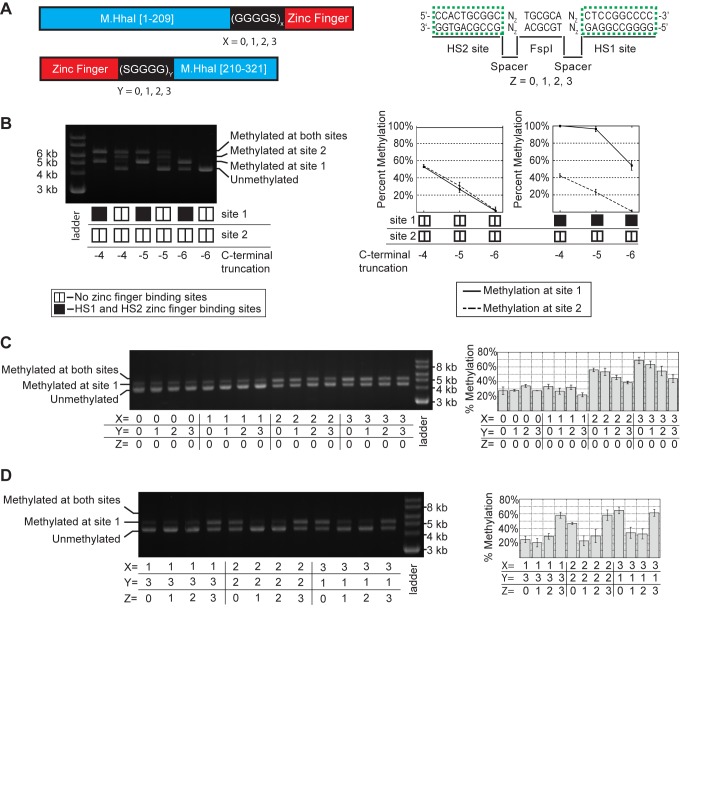


Figure 4: 

**Figure:**
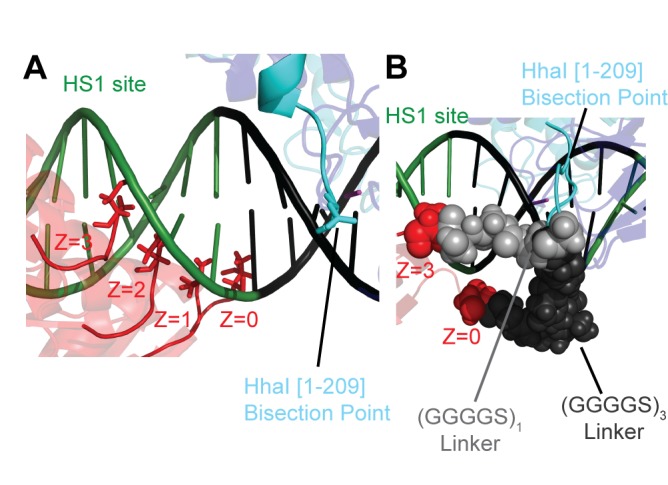


Figure S1: 

Model S1: 
