# Supplementary figures and images for: Targeted DNA Methylation Using an Artificially Bisected M.HhaI Fused to Zinc Fingers
Source: PLoS One. 2012 Sep 11;7(9):e44852. doi: 10.1371/journal.pone.0044852 (PMC3439449; doi:10.1371/journal.pone.0044852)

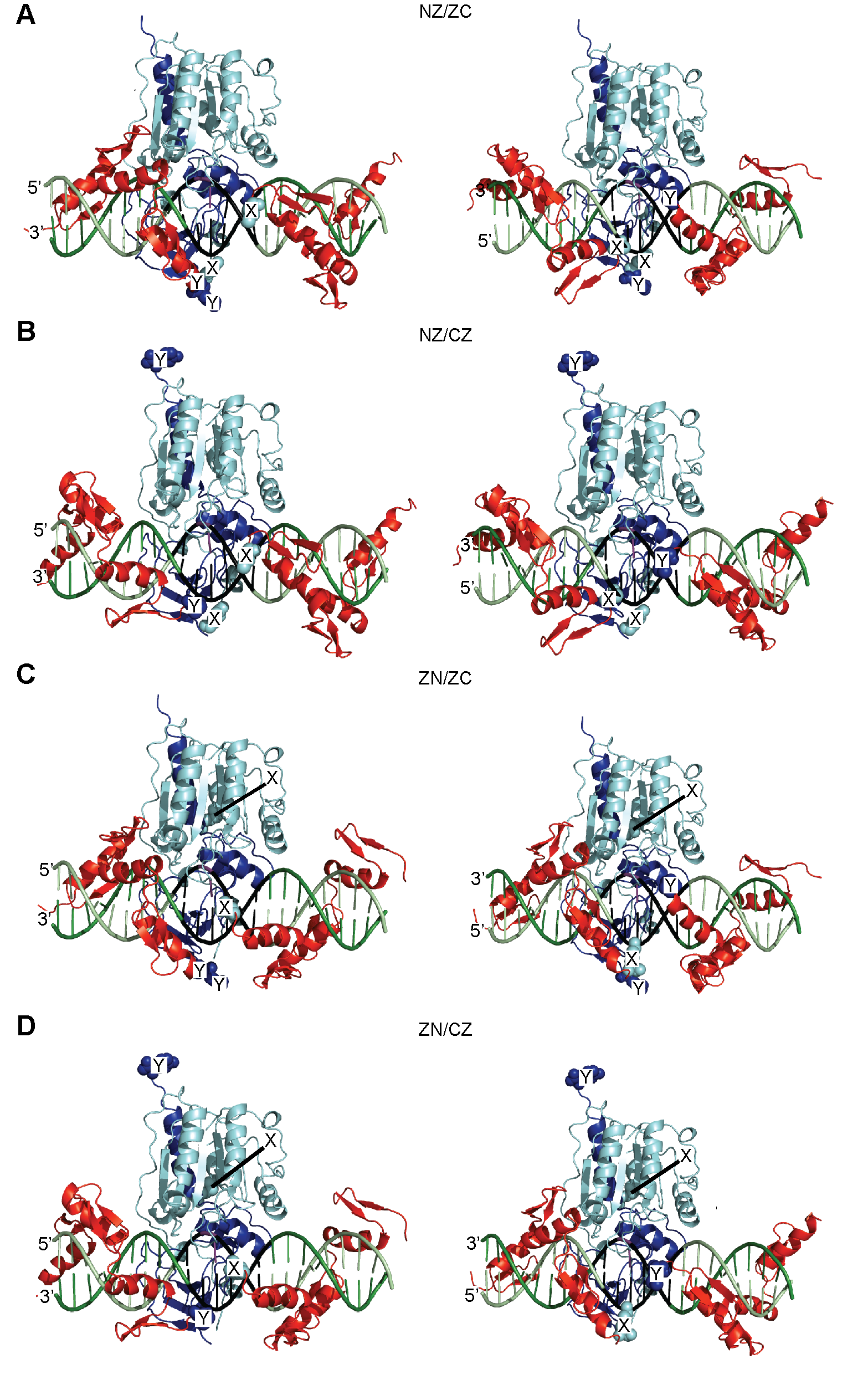

Supplement: Figure S1 — (A–D) Models for four possible fusion combinations of M.HhaI fragments and zinc fingers bound to DNA. For each of the four configurations, models were constructed with the methyltransferase positioned to bind the top or the bottom strand relative to the bound zinc fingers (columns 1 and 2). The ‘X’ and ‘Y’ labels indicate the location of the zinc finger and methyltransferase termini that need to be connected via a peptide linker in order for the zinc finger and M.HhaI domains to be bound to DNA as labeled. The ‘X’ label present on zinc finger HS1 termini should be fused to the ‘X’ label on the termini of M.HhaI [1–209]. The ‘Y’ label present on zinc finger HS2 termini should be fused to the ‘Y’ label on M.HhaI [210–326]. For linear representations of the fusion genes and binding sites see Figures 1A and 1B. (TIF) [file pone.0044852.s001.tif]

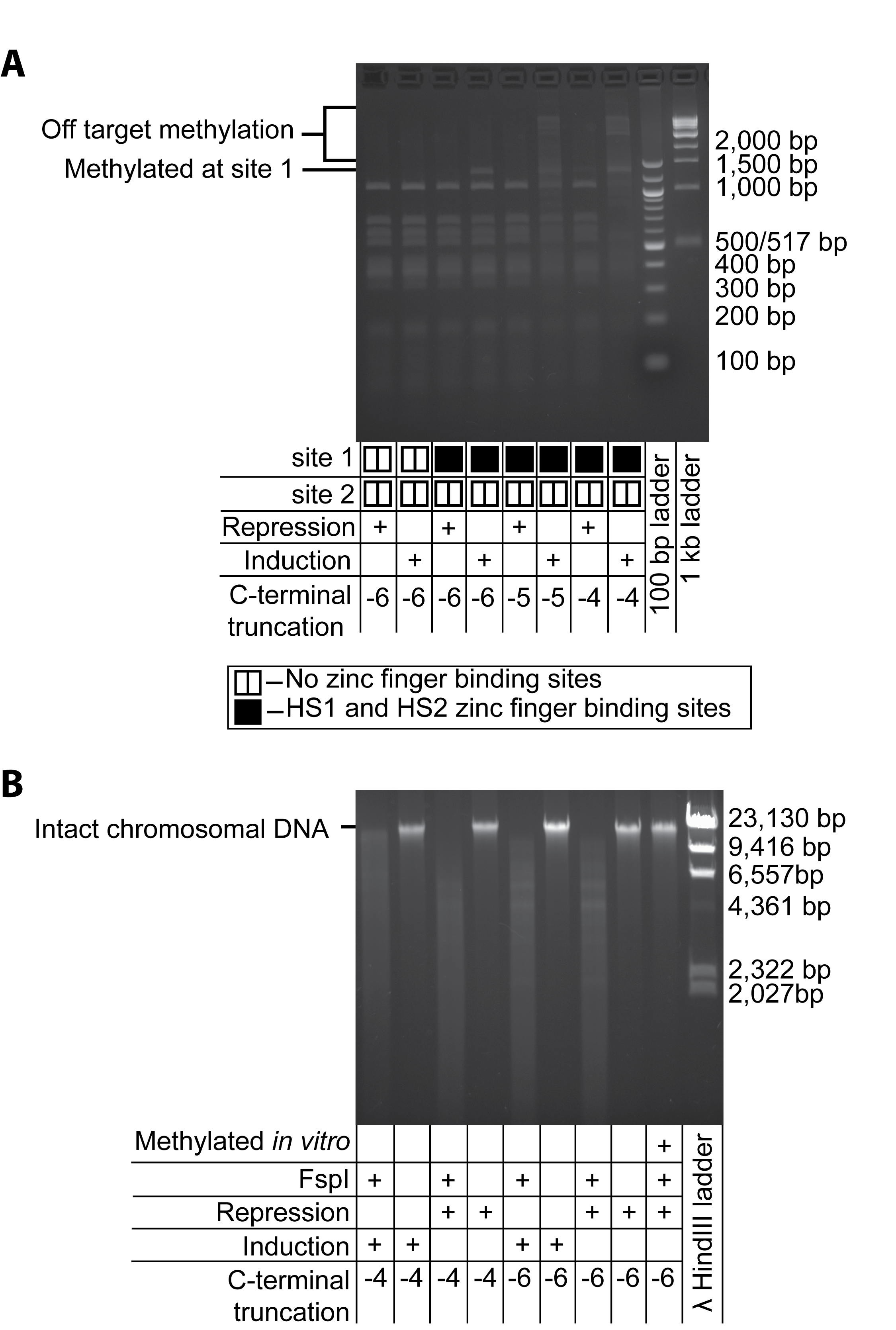

Supplement: Figure S2 — HhaI protection assay of C-terminal truncation variants shown in Figure 3A . (A) Analysis of plasmid DNA. HhaI endonuclease activity is blocked by methylation and one band is indicative of methylation and protection at the target site (site 1). Other, larger bands are indicative of off-target methylation. There are 36 HhaI recognition sites on pDIMN8. Therefore, this assay cannot detect all of the off-target methylation as some bands indicative of off-target methylation may be obscured by other bands in the same lane and some may be too small to observe by this method. (B) Analysis of genomic DNA using FspI digestion. Chromosomal DNA was isolated from cells containing engineered M.HhaI constructs with a 6 or 4 amino acid C-terminal truncation where X = 3, Y = 1, Z = 0. The cells were grown under conditions known to repress or induce methyltransferase fragment expression (see Materials and Methods and Figure 3). The K12 chromosome has over 2000 FspI restriction sites; thus, individual digestion products are not resolvable. For the 6 amino acid-deletion variant this digestion pattern for chromosomal DNA isolated from cells with induced or repressed expression of the engineered methyltransferase is indistinguishable, indicating little to no methylation. However, for the 4 amino acid-deletion variant, induction of the engineered methyltransferase, shifts the digestion pattern toward higher molecular weight bands, which is indicative of some chromosomal methylation. As a control, chromosomal DNA treated with M.HhaI in vitro is protected from FspI digestion. The results show that our targeted methyltransferase (with the 6 amino acid truncation) causes little to no methylation of the chromosome. (TIF) [file pone.0044852.s002.tif]

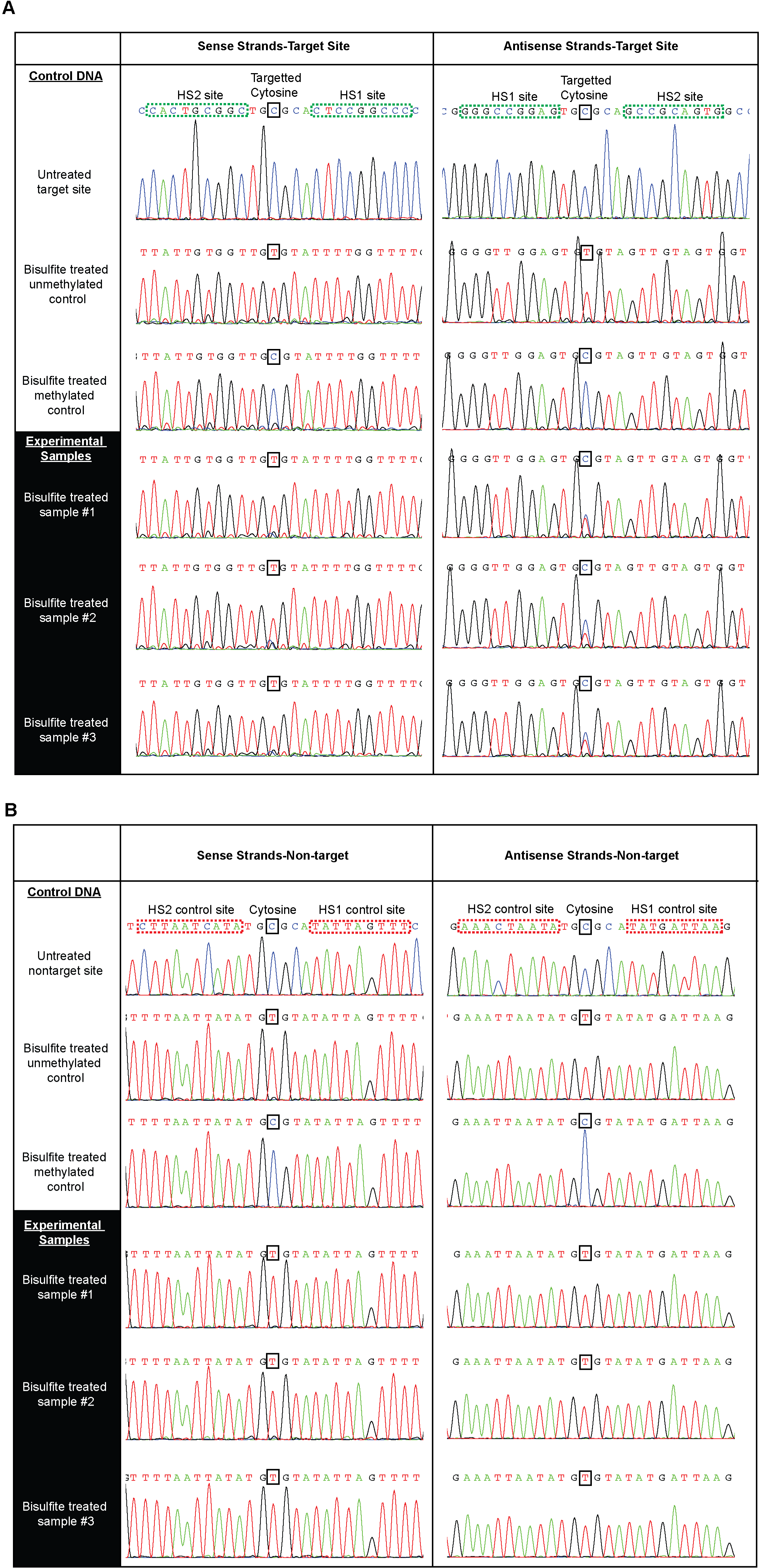

Supplement: Figure S3 — Bisulfite analysis of both strands of the target site and the non-target site. Bisulfite analysis of both strands of (A) the target site and (B) the non-target site. Bisulfite treatment followed by PCR amplification converts unmethylated cytosine bases to thymidine bases. Methylated cytosine residues are protected from such a conversion. The sense strand is defined as the top strand of the target and non-target sites shown in Figure 2D and 2E; the antisense strand is the bottom strand in these figures. Sequenced plasmid DNA, which was not bisulfite-treated is shown in column 1 row 1 of each panel. For both panels, the chromatogram of the antisense strand (column 2 row 1) is the computer-generated reverse complement of the chromatogram in column 1 row 1. DNA in rows 2–6, was treated with the bisulfite reagent, amplified and sequenced as described in Methods S1. The plasmid tested was X = 15, Y = 5, Z = 0 (see Figure 3). Rows 2 and 3 show sequencing results for bisulfite-treated unmethylated and methylated control DNA. Rows 4–6 show sequencing results for bisulfite-treated plasmid DNA from three independent cultures. The chromatograms for the following samples were converted to the reverse complement to simplify the comparison (target site, column 1, rows 4–6). (TIF) [file pone.0044852.s003.tif]
