# Supplementary material for: Targeted DNA Methylation Using an Artificially Bisected M.HhaI Fused to Zinc Fingers
Source: PLoS One. 2012 Sep 11;7(9):e44852. doi: 10.1371/journal.pone.0044852 (PMC3439449; doi:10.1371/journal.pone.0044852)
Supplement: Methods S1 — Supplementary methods section includes modeling details and command-line syntax, HhaI restriction assay, chromosomal restriction assay, and bisulfite sequencing. (DOCX) [file pone.0044852.s004.docx]

**Methods S1**

**Targeted DNA methylation using an artificially bisected M.HhaI fused to zinc fingers.**

Brian Chaikind^1^, Krishna Praneeth Kilambi^2^, Jeffrey J. Gray^2^, and Marc Ostermeier^1,2,^*

^1^Chemistry-Biology Interface Graduate Program, Johns Hopkins University, 3400 N. Charles St., Baltimore, MD 21218-2681 USA

^2^Department of Chemical and Biomolecular Engineering, Johns Hopkins University, 3400 N. Charles St., Baltimore, MD 21218-2681 USA

*To whom correspondence should be addressed. Tel: 410-516-7144; oster@jhu.edu

**Methods S1**

**Modeling details and command-line syntax**

The calculations in the paper were carried out using Rosetta’s developer revision number 46351. All algorithms are also available in Rosetta’s release version 3.4. The Rosetta command-line arguments and scores used for the calculations are as follows.

1. Modeling the zinc fingers

minirosetta.<exe> –database <path_to_rosetta_database>

–run:protocol threading

–in:file:template_pdb 1AAY.pdb

–in:file:alignment 1hs1_.aln

–cm:aln_format general

–frag3 aa1hs1_03_05.200_v1_3

–frag9 aa1hs1_09_05.200_v1_3

–in:file:fasta 1hs1_.fasta

–loops:frag_sizes 9 3 1

–loops:frag_files aa1hs1_09_05.200_v1_3 aa1hs1_03_05.200_v1_3 none

–loops:extended

–loops:build_initial

–loops:remodel quick_ccd

–loops:refine refine_ccd

–out:file:fullatom

–out:nstruct 2000

–out:file:scorefile 1hs1_model.fasc

where 1AAY.pdb is the template structure and 1hs1_.aln is the sequence alignment of target and template

1. Constructing the linker regions (loop building)

loopmodel.<exe> –database <path_to_rosetta_database>
–loops:input_pdb fnl1.pdb
–loops:loop_file fnl1.loop
–loops:remodel perturb_kic
–loops:max_kic_build_attempts 500
–in:file:fullatom

–out:nstruct 100
–out:file:scorefile fnl1.fasc

where fnl1.pdb is the input PDB and fnl1.loop defines the range of loop residues

**HhaI restriction assay**

To ascertain the total methylation status of all 36 HhaI recognition sites ER2267 cells containing methyltransferase fusion constructs were used to inoculate 10 mL of lysogeny broth medium supplemented with 100 μg/mL ampicillin salt. Cells were inoculated in conditions shown to repress or induce methyltransferase gene expression (0.2% w/v glucose for gene repression or 0.2% glucose, 1.0 mM of IPTG and 0.0167% w/v arabinose for gene induction). After 12-14 hours of incubation at 250 rpm and 37˚ C, plasmid DNA was isolated from the cells using QIAprep spin miniprep kit (Qiagen, Valencia, CA, USA).

Plasmid DNA (500 ng) was incubated with 10 units of HhaI in 1X NEB4 and 1 μg/mL BSA at 37˚ C for 2 hours. After digestion, the DNA was electrophoresed in a 2.5% w/v agarose gel in TAE buffer at 90 V for 50 minutes at room temperature. Images were captured using the Molecular Imager XRS Gel Doc system with Quantity One software.

**Chromosomal Restriction Assay**

ER2267 cells containing variants with X=3, Y=1, and Z=0, a C-terminal truncation of 6 or 4 amino acids, and zinc finger binding sequences at site 1, were grown under conditions known to induce or repress expression of the heterodimeric methyltransferase (see Materials and Methods). Chromosomal DNA was isolated using the Sigma’s GenElute Bacterial Genomic Kit according to the manufacturer’s instructions (Sigma-Aldrich, St. Louis, Missouri , USA). Chromosomal DNA was electrophoresed on a 0.8% w/v agarose gel in TAE buffer at 90V for 35 min. to separate genomic DNA from plasmid DNA. Chromosomal DNA was isolated from the gel and purified using Invitrogen’s PureLink Quick Gel Extraction Kit (Invitrogen Carlsbad, CA, USA). DNA was further purified by ethanol precipitation as described in Sambrook and Russel [1]. The global level of methylation on the chromosome was assessed by incubating 250 ng of DNA with 5 U of FspI in 1X NEB4 for 1 hr at 37°C. A methylated control was prepared by incubating 250 ng of chromosomal DNA at 37°C for 1 hour with 12.5 U of M.HhaI in 1X HhaI methylase reaction buffer and 80 μM S-adenosyl-methionine, heat killing the reaction at 65°C for 20 minutes and then incubating the reaction with 5 U of FspI in 1X NEB4 for 1 hr at 37°C. (New England Biolabs, Ipswich, MA, USA). DNA was electrophoresed on a 1% w/v agarose gel in TAE at 90V for 70 minutes. Images were captured using the Molecular Imager XRS Gel Doc system with Quantity One software.

**Bisulfite Sequencing**

Unmethylated pDIMN8 plasmid was obtained by inoculating cells in 10 mL of lysogeny broth under conditions that repress gene expression, as described above. Methylated controls were obtained by incubating 8 μg of the unmethylated plasmid at 37°C for 1 hour with 50 units of M.HhaI (New England Biolabs, Ipswich, MA, USA) in 1X HhaI methylase reaction buffer supplemented with 32 mM S-adenosylmethionine. DNA was purified using the Zymo Clean and Concentrator kit according to manufacturers instructions (Zymo Research Corporation, Irvine, CA, USA).

For the experimental samples, ER2267 cells containing pDIMN8 plasmids encoding for methyltransferase fragments, were grown under conditions that induce expression, in triplicate, as described above.

DNA was isolated from cells using QIAprep spin miniprep kit and digested with 20-40 units of NcoI at 37˚ C for 1-2 hours (Qiagen, Valencia, CA). Linearized DNA was purified using the Zymo Clean and Concentrator kit according to manufacturers instructions.

DNA was then treated with bisulfite reagent and purified using the EZ DNA Methylation-Gold Kit according to manufacturers instructions (Zymo Research Corporation, Irvine, CA, USA). Individual strands from site 1 and site 2 were then amplified with a set of unique primers, using One Taq Hot Start DNA Polymerase (New England Biolabs, Ipswich, MA, USA). PCR amplified DNA was purified using the Zymo Clean and Concentrator kit. Purified, PCR amplified DNA, 10 ng, was directly sequenced by Genewiz (South Plainfield, NJ, USA). The heights of trace files in Figure S3 were adjusted to aid in direct comparison.

1. Sambrook J, Russel, DW (2001) Molecular Cloning: A Laboratory Manual. 3rd ed. Cold Spring Harbor, NY: Cold Spring Harbor Laboratory Press. pp A8.12-A8.16.
