# Supplementary material for: Targeted DNA Methylation Using an Artificially Bisected M.HhaI Fused to Zinc Fingers
Source: PLoS One. 2012 Sep 11;7(9):e44852. doi: 10.1371/journal.pone.0044852 (PMC3439449; doi:10.1371/journal.pone.0044852)
Supplement: Models S1 — PDB files of all models in a.zip file format. (ZIP) [file pone.0044852.s005.zip › Models_S1/description_of_PDB_models.pdf]

Description of PDB files found in the supplementary files of "Targeted DNA methylation using an artificially bisected M.HhaI fused to zinc fingers."

FInal_NZ_ZC_model.pdb
Description: The model of NZ/ZC shown in figure 1C. M.HhaI is bound to the bottom strand of the target site as shown in figure 2D. M.HhaI [1-209] and M.HhaI [210-326] are connected to zinc fingers by 15 and 5 amino acid linkers, respectively.

NZ_ZC.1.pdb
Description: The model of NZ/ZC shown in column 1 of supplemental figure 1A. M.HhaI is bound to the top strand of the target site as shown in figure 2D.

NZ_ZC.2.pdb
Description: The model of NZ/ZC shown in column 2 of supplemental figure 1A. M.HhaI is bound to the bottom strand of the target site as shown in figure 2D.

NZ_CZ.1.pdb
Description: The model of NZ/CZ shown in column 1 of supplemental figure 1B. M.HhaI is bound to the top strand of the target site as shown in figure 2D.

NZ_CZ.2.pdb
Description: The model of NZ/CZ in column 2 of supplemental figure 1B. M.HhaI is bound to the bottom strand of the target site as shown in figure 2D.

ZN_ZC.1.pdb
Description: The model of ZN/ZC shown in column 1 of supplemental figure 1C. M.HhaI is bound to the top strand of the target site as shown in figure 2D.

ZN_ZC.2.pdb
Description: The model of ZN/ZC shown in column 2 of supplemental figure 1C. M.HhaI is bound to the bottom strand of the target site as shown in figure 2D.

ZN_CZ.1.pdb
Description: The model of ZN/CZ shown in column 1 of supplemental figure 1D. M.HhaI is bound to the top strand of the target site as shown in figure 2D.

ZN_CZ.2.pdb
Description: The model of ZN/CZ shown in column 2 of supplemental figure 1D. M.HhaI is bound to the bottom strand of the target site as shown in figure 2D.

NZ_ZC.spacer.0.pdb
NZ_ZC.spacer.1.pdb
NZ_ZC.spacer.2.pdb
NZ_ZC.spacer.3.pdb
Description: The models shown in Figure 4. The zinc finger binding sites separated from the internal FspI site by 0,1,2, or 3 bases respectively. In these models, M.HhaI is bound to the bottom strand of the target site as shown in figure 2D.
