# Supplementary figures and images for: Correction: Targeted DNA Methylation Using an Artificially Bisected M.HhaI Fused to Zinc Fingers
Source: PLoS One. 2014 Jan 29;9(1):10.1371/annotation/b1f48851-2dd7-438f-938e-6bac0b4d8c94. doi: 10.1371/annotation/b1f48851-2dd7-438f-938e-6bac0b4d8c94 (PMC3906384; doi:10.1371/annotation/b1f48851-2dd7-438f-938e-6bac0b4d8c94)

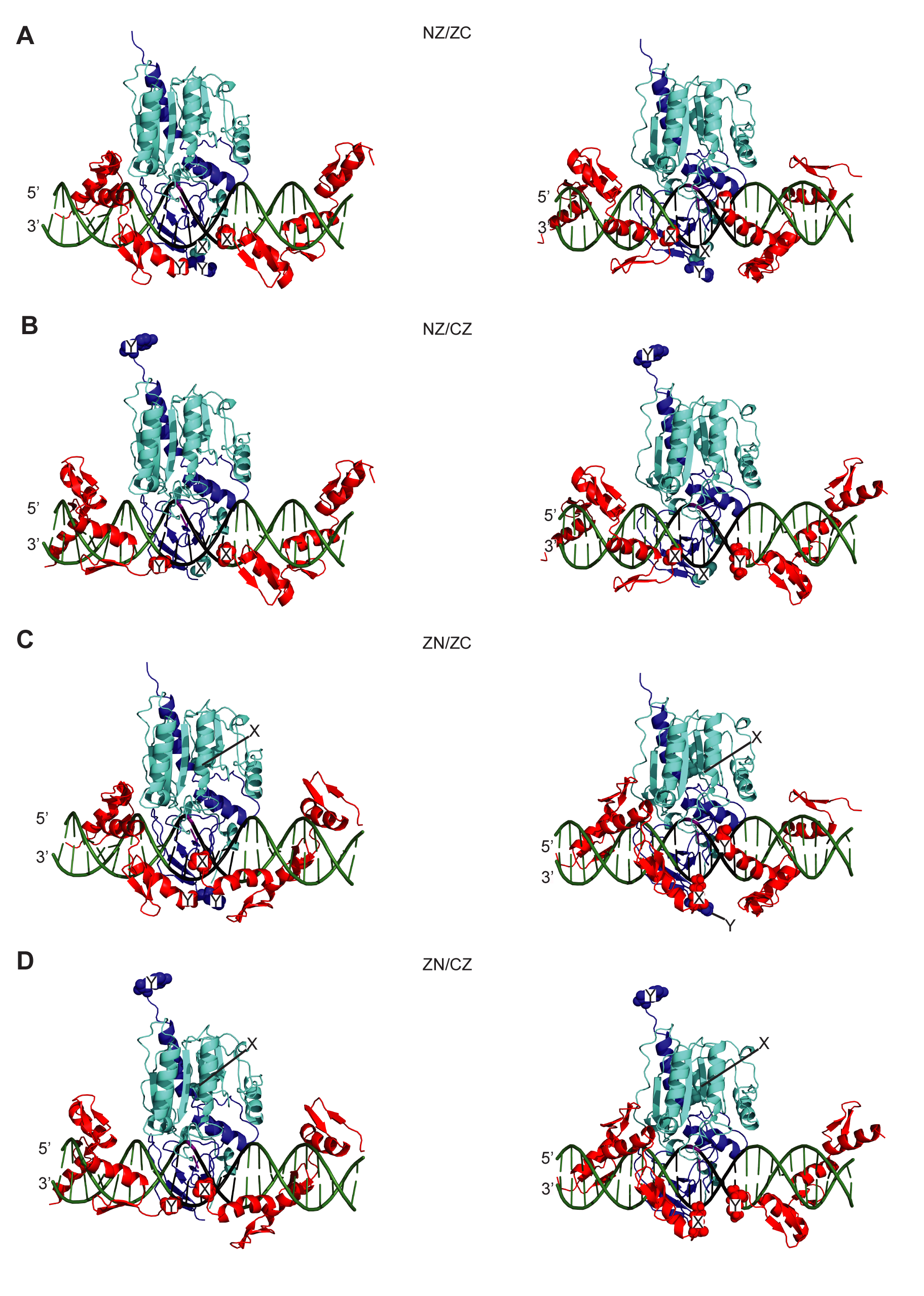

Supplement: Supplementary file 1 [file pone.b1f48851-2dd7-438f-938e-6bac0b4d8c94.s001.tif]
